# Keratinocyte‐Derived Glucocorticoids Maintain Immune Balance During Transient Skin Barrier Perturbation

**DOI:** 10.1111/all.16613

**Published:** 2025-06-05

**Authors:** Truong San Phan, Rebekka Lambrecht, Verena M. Merk, Alice Wiedmann, Pascale Zwicky, Burkhard Becher, Thomas Brunner

**Affiliations:** ^1^ Biochemical Pharmacology, Department of Biology University of Konstanz Konstanz Germany; ^2^ Inflammation Research, Institute of Experimental Immunology University of Zurich Zurich Switzerland

**Keywords:** animal models, barrier, dermatology, epithelium, immune tolerance, inflammation, keratinocytes

**Abbreviations**
BD: barrier disruption
Cyp11b1: 11β‐hydroxylase
GCs: glucocorticoids
IntB: intact barrier
Krt14: Keratin 14
RNA‐seq: RNA‐sequencing
dLNs: draining lymph nodes
DAMPs/PAMPs: damage‐ and pathogen‐associated molecular patterns

To the Editor,

Epithelial barrier integrity and local immune regulation are fundamental to health, preventing pathogen entry and limiting inflammatory responses. Their dysregulation is increasingly recognized as a key driver of chronic inflammatory and allergic diseases [1]. Endogenous glucocorticoids (GCs) are potent anti‐inflammatory steroid hormones, representing a cornerstone in inflammatory disease regulation. Beyond adrenal synthesis, keratinocytes also locally produce GCs in the skin [2, 3, 4]. Deleting the GC‐producing enzyme 11β‐hydroxylase (*Cyp11b1*) in keratinocytes has been shown to exacerbate inflammatory skin conditions [2]. To investigate in vivo keratinocyte‐derived GC deficiency in conditions with and without skin barrier disruption (BD), we compared two models to induce *Cyp11b1* knockout (KO) in keratinocytes using *Krt14*‐*CreER* ^*TAM*^ x *Cyp11b1* ^*L2*/*L2*^ mice and control floxed mice: local topical tamoxifen application with mild skin barrier disruption (BD) (BD‐KO for KO mice, BD‐L2/L2 for floxed control mice), or i.p. tamoxifen injection, preserving the intact barrier integrity in control and KO mice (IntB‐L2/L2, IntB‐KO) (Figure 1A).

**FIGURE 1:**
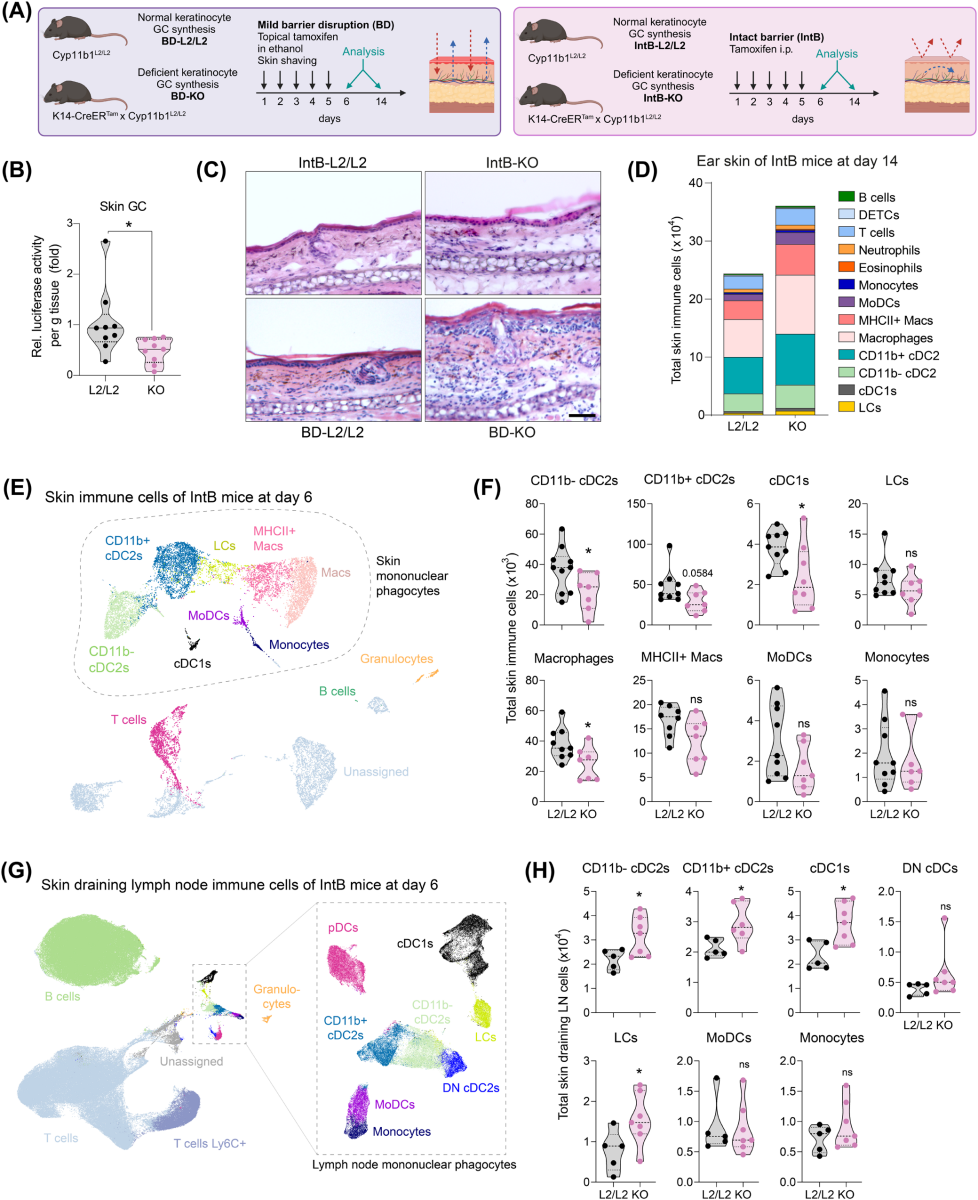
Keratinocyte‐derived GC ablation triggers APC emigration to skin dLNs. (A) Experimental timeline for i.p. or topical tamoxifen application in floxed (L2/L2) and *Krt14*‐Cre + (KO) mice for keratinocyte‐specific *Cyb11b1* deletion in KO mice. Topical administration includes mild barrier disruption (BD), while i.p. injection preserves an intact skin barrier (IntB). (B) Glucocorticoid receptor response element luciferase reporter assay of ex vivo skin culture supernatants from IntB mice at day 6 (*n* = 9, Student's *t*‐test). (C) Representative hematoxylin and eosin‐stained day 14 ear sections. Scale bar, 50 μm. (D) Stacked bar showing total ear skin immune cell counts from IntB mice after 14 days (*n* = 6). DETCs, dendritic epidermal T cells; cDCs, conventional dendritic cells; LCs, Langerhans cells, MoDCs, monocyte‐derived DCs; Macs, macrophages. (E–H) Flow cytometry‐derived UMAP of skin and draining lymph node (dLN) immune cells and total cell counts from i.p.‐induced IntB‐KO mice at day 6. DN, double negative. Dots indicate individual mice (*n* = 7–9 skin, *n* = 5–7 dLNs). Straight lines represent mean.

As previously reported [2], abrogation of keratinocyte GC synthesis with mild BD resulted in the development of a spontaneous skin inflammation after 14 days (Figure 1C). Despite similar keratinocyte‐specific *Cyp11b1* deletion and diminished skin GC levels (Figure 1B and Figure S1A) [2], i.p.‐induced IntB‐KOs showed moderately increased skin immune cells, without developing spontaneous skin inflammation (Figure 1C,D and Figure S1B,C). Similarly, their skin draining lymph nodes (dLNs) were not altered in proportion and lymphocyte function, and did not demonstrate increased immune cell activation (Figure S1D–F).

Furthermore, antigen‐presenting cells (APCs) in skin and dLN of IntB‐KOs were compared with IntB‐L2/L2 control mice to assess potential differences in peripheral immune priming at an earlier time point (day 6). The combination of keratinocyte‐derived GC deficiency and skin barrier breach resulted in increased skin APC emigration and immune priming in BD‐KO, which are more exposed to Toll‐like receptor and cytokine signaling (Phan et al. 2021). In contrast, and in the absence of those signals, i.p.‐induced IntB‐KOs also promoted skin APC emigration but lacked inflammatory priming, consistent with the missing inflammatory phenotype compared to BD‐KOs (Figure 1E–H and Figure S1G–I).

Loss of keratinocyte GC synthesis in naive, non‐irritated skin resulted in increased APC emigration, but failed to induce skin inflammation compared to topically induced BD‐KO mice. Since this indicates that local regulation may be maintained by keratinocytes, we elucidated the autoregulatory effects of keratinocyte‐derived GCs in the absence or presence of BD. Keratinocytes from topical‐induced BD‐KOs and BD‐L2/L2 controls as well as from i.p.‐induced IntB‐KO and IntB‐L2/L2 were isolated and bulk‐sorted for RNA sequencing (Figure 2A,B, S2A‐C). Only BD‐KO‐derived keratinocytes upregulated inflammatory and cell death‐associated pathways (Figure 2C–E, Figure S2D and Table S3) consistent with the inflammatory skin phenotype (Figure 1C) [2]. In contrast, KO keratinocytes from intact skin barriers exhibited non‐significant cytokine‐ and stress‐related processes, downregulated inflammation, and elevated TGF‐β pathway, suggesting a counterbalanced response (Figure 2D,E). Specifically, BD‐KO‐derived keratinocytes demonstrated increased pro‐apoptotic and DNA damage response genes, while IntB‐KO keratinocytes upregulated anti‐apoptotic genes (Figure 2E). Only combined GC loss and skin BD induced pro‐inflammatory and interferon‐stimulated gene expression (Figure 2E) [2], whereas i.p.‐induced IntB‐KO keratinocytes upregulated anti‐inflammatory genes (Figure 2E). Notably, increased key GC‐responsive genes (*Tsc22d3, Rasd1*) suggested elevated adrenal GC synthesis in the IntB‐KO condition. Unlike the locally restricted, topical‐induced BD‐KOs^2^, serum GCs and IL‐6 levels were indeed elevated in the i.p.‐induced body‐wide keratinocyte *Cyp11b1* IntB‐KOs (Figure 2F,G), suggesting a systemic response and compensatory adrenal GC release to the keratinocyte‐deficient GC synthesis, which warrants further investigation and validation (Figure 2H).

**FIGURE 2:**
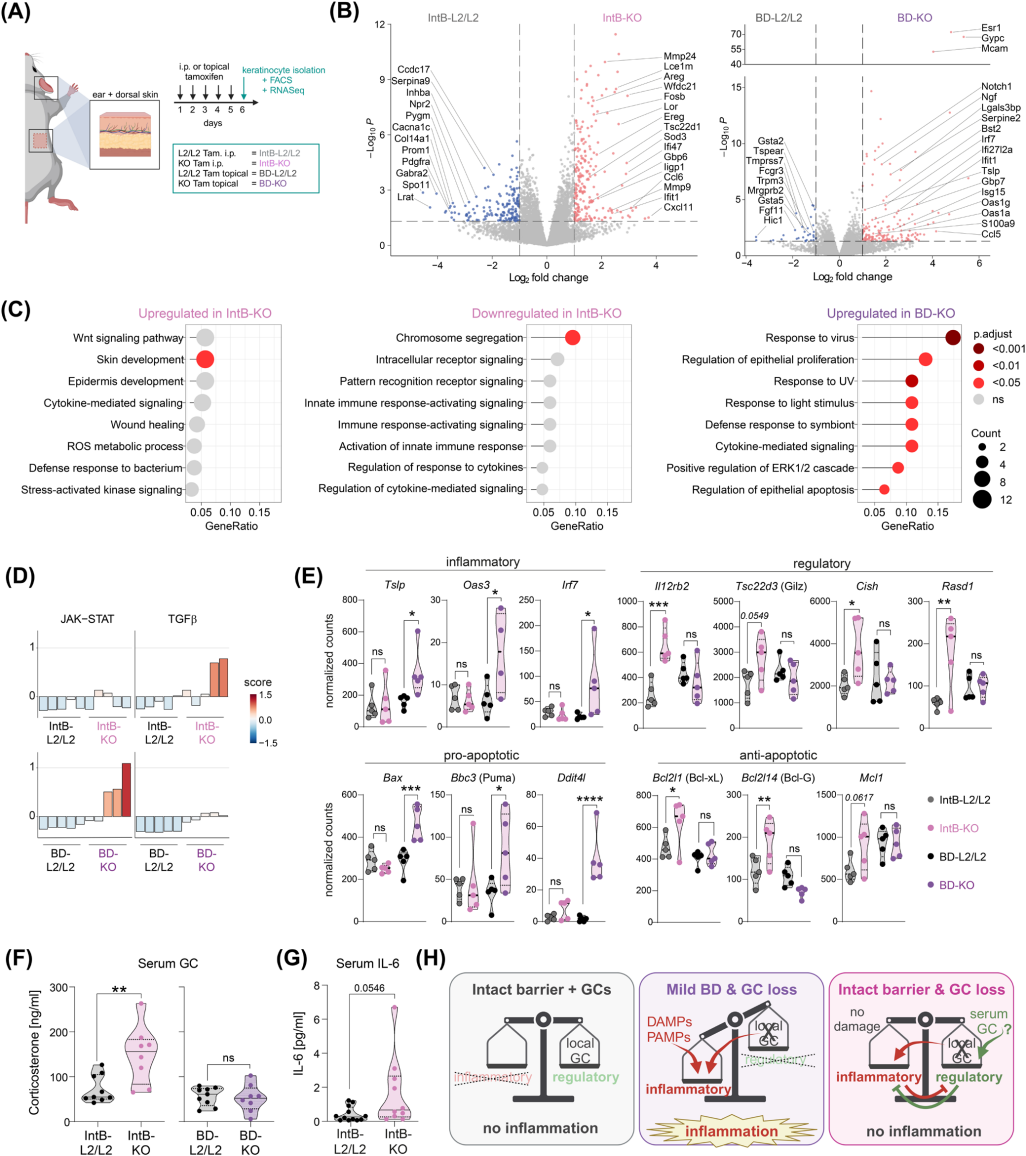
Skin GC deficiency provokes inflammatory signaling in keratinocytes. (A) Experimental timeline for in vivo keratinocyte‐specific *Cyb11b1* deletion in *Krt14*‐Cre + (KO) and floxed control (L2/L2) mice via i.p. tamoxifen injection (ntact skin barrier, IntB) or topical tamoxifen application (mild skin barrier disruption, BD), followed by isolation, bulk‐sorting and RNA‐sequencing of keratinocytes on day 6 (*n* = 5). (B) Volcano plot of differentially expressed genes (DEGs) between L2/L2 and KO from IntB or BD keratinocytes (significant: *p*‐value < 0.05, log_2_ fold change < −1 or > 1). (C) Lollipop plot depicts enrichment of up−/downregulated DEGs (B) in the gene ontology ‘Biological processes‘in IntB‐KO and BD‐KO keratinocytes compared to their respective controls. (D) JAK–STAT and TGFβ PROGENy pathway activity scores of pairwise comparison (KO vs. L2/L2 keratinocytes) for each condition. (E) Violin plot showing normalized gene expression counts of L2/L2 and KO keratinocytes from IntB and BD conditions. Statistical test performed using two‐way ANOVA with Sidak's multiple comparison test. (F, G) Serum GC concentration (F) measured by GC response element reporter assay (left) from IntB‐derived mice and GC radioimmunoassay (right) (Phan et al., *Sci Adv*. 2021) [2] from BD mice (*n* = 8–9 mice). (G) Serum IL‐6 concentration from IntB mice (*n* = 9–12). Statistical significance was determined using unpaired two‐tailed Student's *t*‐test. (H) Graphical summary of synergistic effect between skin BD and loss of keratinocyte GC production leading to skin inflammation. Created with BioRender.com.

Our findings underscore the importance of keratinocyte‐derived GCs in skin immune regulation, acting synergistically with an intact epithelial barrier. Only disruption of both barrier integrity and local keratinocyte‐specific GC synthesis results in skin inflammation and systemic stress responses, associated with heightened susceptibility to inflammatory and allergic skin conditions. These results align with well‐documented studies about the role of epithelial barriers, resp. GC in skin homeostasis [5, 6]. The relevance of local GC synthesis and epithelial barrier integrity for the skin may also apply to other barrier tissues, such as the intestine and the lung, as these organs also produce GC and similarly require sensitive tissue‐immune regulation.

## Author Contributions

Conceptualization: T.S.P., T.B.; Formal analysis: R.L., T.S.P.; Funding acquisition: T.S.P., T.B.; Investigation: R.L., T.S.P., V.M.M., A.W., P.Z.; Resources: T.B., B.B.; Supervision: T.B.; Writing – Original Draft Preparation: RL; Writing – Review and Editing: R.L., T.S.P., T.B.

## Conflicts of Interest

The authors declare no conflicts of interest.

## Supporting information


**Figure S1.** Ablation of skin GC synthesis by i.p. administration of tamoxifen does not prime skin draining lymph nodes immune cells.
**Figure S2.** Differences between keratinocyte GC deficiency in IntB and BD mice.

## Data Availability

The data that support the findings of this study are openly available in GEO at https://www.ncbi.nlm.nih.gov/geo/query/acc.cgi?acc=GSE271160, reference number GSE271160.
