# Supplementary material for: Keratinocyte‐Derived Glucocorticoids Maintain Immune Balance During Transient Skin Barrier Perturbation
Source: Allergy. 2025 Jun 5;80(8):2384–7. doi: 10.1111/all.16613 (PMC12368758; doi:10.1111/all.16613)
Supplement: Supplementary file 1 — Figure S1. Ablation of skin GC synthesis by i.p. administration of tamoxifen does not prime skin draining lymph nodes immune cells. Figure S2. Differences between keratinocyte GC deficiency in IntB and BD mice. [file ALL-80-2384-s001.doc]

**Supplementary Figures, Materials and Methods**

**
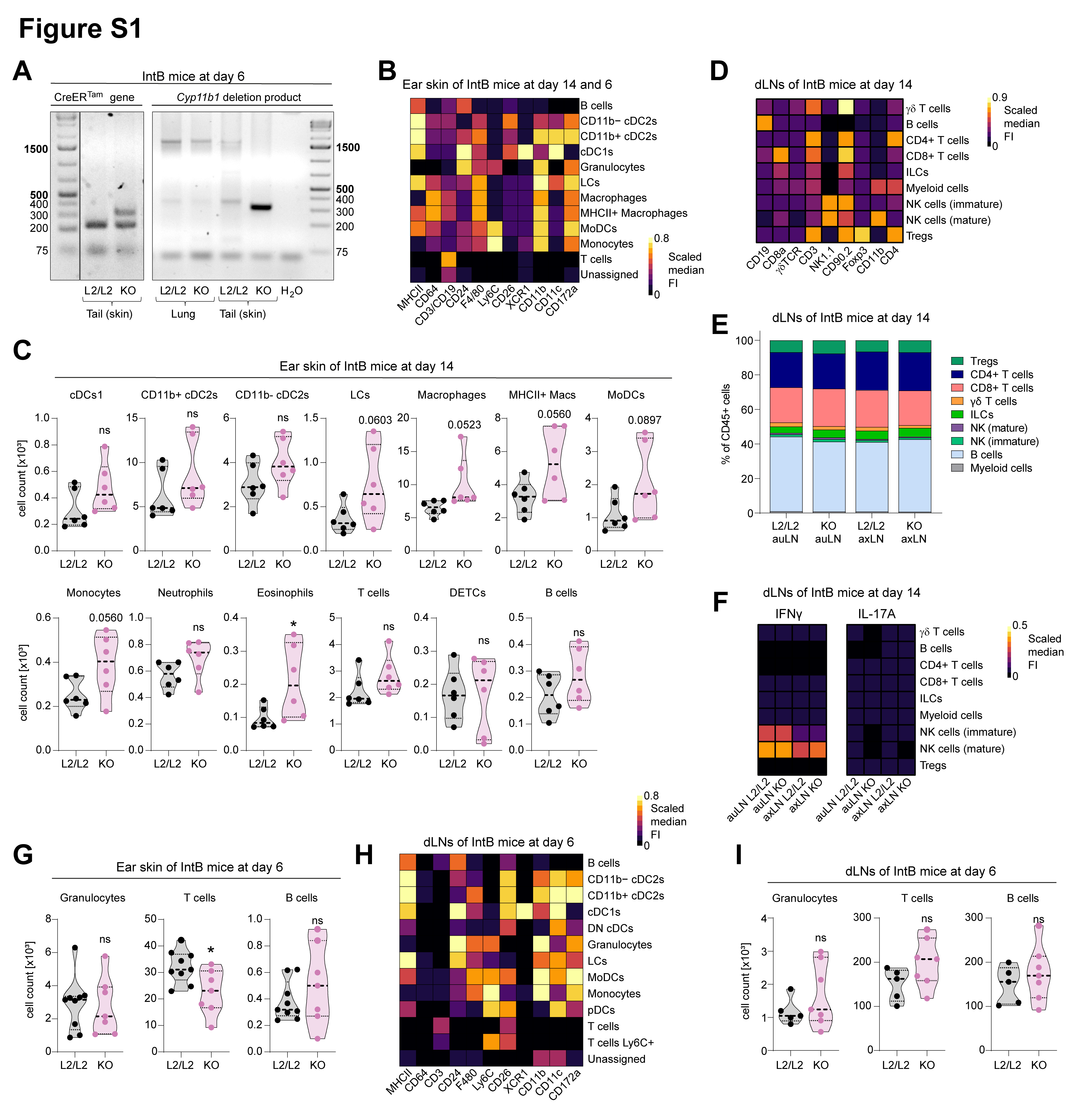
**

**Figure S1: Ablation of skin GC synthesis by i.p. administration of tamoxifen does not prime skin draining lymph nodes immune cells.**

**(A)** Agarose gel electrophoresis of PCRs with genomic DNA isolated from tail or lung tissue of *K14*-*CreER^Tam^* x *Cyp11b1^L2^*/*^L2^* knockout (KO) and *Cyp11b1^L2^*/*^L2^* (L2/L2) mice that were treated for 5 days with tamoxifen via i.p. injection (with intact skin barrier, IntB mice) and sacrificed on day 6. Numbers on the left indicate size in base pairs (bp). (Left) CreER^Tam^ gene PCR shows presence of Cre recombinase gene with an amplicon size of 320 bp. Myogenin PCR product (250 bp) served as internal control. (Right) *Cyp11b1* deletion PCR amplicon at 349 bp in Cre^+^ mice (KO) depicts successful *in vivo* recombination while a 1765 bp amplicon represents wild-type *Cyp11b1*.

**(B)** Heatmap with scaled median fluorescence intensity (FI) of flow cytometry markers over FlowSOM-annotated clusters from ear skin immune cells derived from IntB-L2/L2 and -KO mice at day 6 and 14.

**(C)** Total skin immune cell counts as in (B) from ear skin of IntB-L2/L2 and -KO mice on day 14. Dots indicate individual mice (n=6, Student’s t test). DETCs, dendritic epidermal T cells. Bold dashed line represents median and regular dashed lines show quartiles.

**(D)** Heatmap with scaled median FI of flow cytometry markers over FlowSOM-annotated clusters from skin draining lymph node (dLN) cells derived from IntB-L2/L2 and -KO mice at day 14.

**(E)** Stacked bar plot depicting skin dLN cell proportions of IntB-L2/L2 and -KO mice at day 14. Bars display mean of individual mice (n=6).

**(F)** Heatmap with scaled median FI of intracellular IL-17A and IFN-γ expression of indicated immune cells from auricular (au) and axially (ax) LNs of IntB-L2/L2 and -KO mice on day 14. Cells were *ex vivo* stimulated with PMA/Ionomycin before flow cytometry analysis.

(**G**) Total cells of ear skin immune cell populations from IntB-L2/L2 and -KO mice at day 6. Dots indicate individual mice (n=7-9, Student’s t test). Bold dashed line represents median and regular dashed lines show quartiles.

**(H)** Heatmap with scaled median FI of flow cytometry markers over FlowSOM-annotated clusters from skin dLN immune cells derived from IntB-L2/L2 and -KO mice at day 6.

**(I)** Total skin dLN immune cell counts from IntB-L2/L2 and -KO mice at day 6. Dots indicate individual mice (n=5-7). Bold dashed line represents median and regular dashed lines show quartiles.

cDCs, conventional dendritic cells; LCs, Langerhans cells; MoDCs, monocyte-derived DCs; DN, double negative (CD11b- CD172a-).LCs, Langerhans cells; ILCs, innate lymphoid cells; NK, natural killer cells.

**
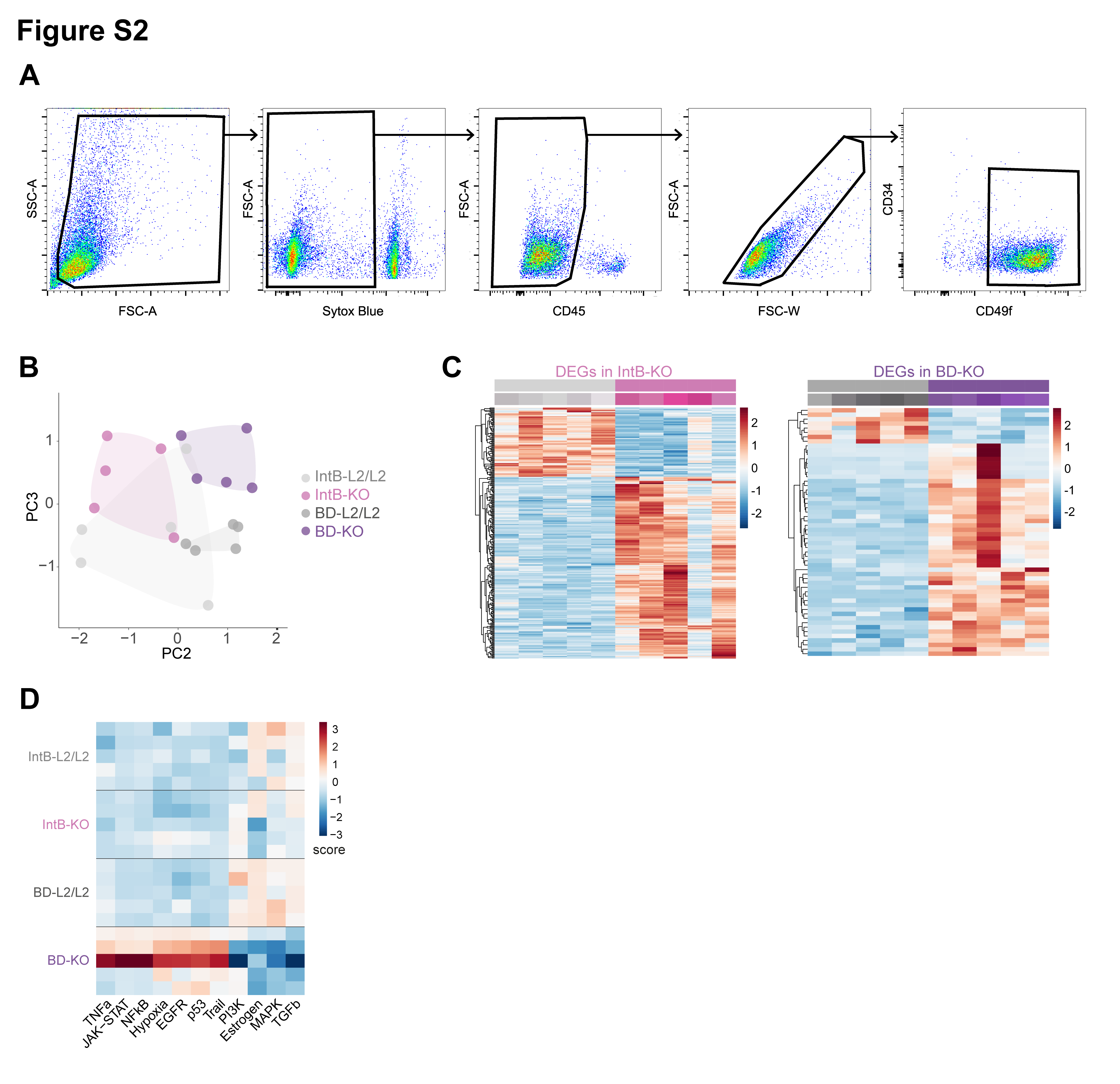
**

**Figure S2: Differences between keratinocyte GC deficiency in IntB and BD mice.**

**(A)** Gating strategy for sorting ear and dorsal skin keratinocytes prior to RNA sequencing. Live keratinocytes were identified as Sytox Blue-negative, CD45-negative single cells positive for CD49f.

**(B)** Principal component analysis (PCA) plot of keratinocyte bulk RNA sequencing data. Each dot represents an individual mouse (n=5 mice per group). Keratinocytes were isolated from *K14*-*CreER^Tam^ x Cyp11b1^L2^*/*^L2^* knockout (KO) and *Cyp11b1^L2^*/*^L2^* (L2/L2) control mice that received tamoxifen either by i.p. injection with intact skin barrier (IntB) or via topical administration including mild barrier disruption (BD).

**(C)** Heatmap of z-score normalized differentially expressed genes (DEGs) between KO and L2/L2 control keratinocytes from IntB and BD conditions.

(**D**) Heatmap of PROGENy pathway activity scores of normalized counts combined from all conditions.

**Supplementary Tables**

Table S1: Flow cytometry antibodies for skin and dLNs analysis at Day 6

| **Antigen target** | **Fluorophore** | **Provider** | **Cat. Number** |
| --- | --- | --- | --- |
| CD24 | BV421 | BioLegend | 101825 |
| F4/80 | BV605 | BioLegend | 123133 |
| CD172a | PerCP-eFluor710 | eBioscience | 46-1721-80 |
| CD26 | FITC | BioLegend | 137805 |
| Ly-6C | BV785 | BioLegend | 128041 |
| XRC1 | PE | BioLegend | 148203 |
| CD11b | PE-Cy7 | BioLegend | 101216 |
| CD64 | Alexa Fluor 647 | BioLegend | 139321 |
| CD45 | Alexa Fluor 700 | BioLegend | 103128 |
| CD11c | PE eFluor610 | eBioscience | 61-0114-82 |
| MHCII | APC eFluor 780 | eBioscience | 47-5321-82 |
| CD19 | eFluor 506 | eBioscience | 69-0193-80 |
| CD3 | eFluor 506 | eBioscience | 69-0032-80 |

Table S2: Flow cytometry antibodies for dLNs analysis at Day 14

| **Antigen target** | **Fluorophore** | **Provider** | **Cat. Number** |
| --- | --- | --- | --- |
| CD45 | FITC | eBioscience | 11-0451-85 |
| CD11b | PE-Cy7 | BioLegend | 101216 |
| CD3 | BV605 | BioLegend | 100237 |
| CD4 | PE/Dazzle594 | BioLegend | 100455 |
| CD8 | BV421 | BioLegend | 100737 |
| NK1.1 | BV711 | BioLegend | 108745 |
| γδTCR | BV510 | BioLegend | 118131 |
| CD19 | APC-Fire750 | BioLegend | 115557 |
| CD90.2 | BV786 | BioLegend | 105331 |
| FoxP3 | PE | eBioscience | 12-5773-80 |
| IFN-y | APC | BioLegend | 505809 |
| IL-17A | PerCP-Cy5.5 | eBioscience | 45-7177-82 |

Table S3: Top 40 Biological Processes (BPs) form gene ontology (GO) analysis,
red colored marks the selected BPs used in main Figure 2D

| **No** | **Up-regulated in IntB-KO** | **Down-regulated IntB-KO** | **Up-regulated in BD-KO** |
| --- | --- | --- | --- |
| 1 | skin development | chromosome segregation | response to virus |
| 2 | epidermis development | mRNA processing | regulation of epithelial cell proliferation |
| 3 | Wnt signaling pathway | nuclear division | response to UV |
| 4 | cell-cell signaling by wnt | intracellular receptor signaling pathway | defense response to virus |
| 5 | cytokine-mediated signaling pathway | negative regulation of neuron projection development | response to light stimulus |
| 6 | positive regulation of proteolysis | negative regulation of cell projection organization | defense response to symbiont |
| 7 | regulation of peptidase activity | pattern recognition receptor signaling pathway | response to radiation |
| 8 | organic hydroxy compound biosynthetic process | innate immune response-activating signaling pathway | cytokine-mediated signaling pathway |
| 9 | regulation of Wnt signaling pathway | protein polyubiquitination | regulation of cell growth |
| 10 | regulation of endopeptidase activity | activation of innate immune response | gland development |
| 11 | wound healing | nuclear chromosome segregation | gland morphogenesis |
| 12 | glycerolipid metabolic process | glycerolipid metabolic process | negative regulation of cell growth |
| 13 | developmental maturation | positive regulation of innate immune response | positive regulation of ERK1 and ERK2 cascade |
| 14 | positive regulation of kinase activity | regulation of developmental growth | negative regulation of growth |
| 15 | regulation of cell growth | positive regulation of response to biotic stimulus | developmental growth involved in morphogenesis |
| 16 | cellular component disassembly | regulation of translation | regulation of cellular response to growth factor stimulus |
| 17 | reactive oxygen species metabolic process | immune response-activating signaling pathway | reproductive structure development |
| 18 | Golgi vesicle transport | immune response-regulating signaling pathway | regulation of ERK1 and ERK2 cascade |
| 19 | anatomical structure maturation | cytokine-mediated signaling pathway | reproductive system development |
| 20 | Ras protein signal transduction | regulation of cell growth | negative regulation of locomotion |
| 21 | response to salt | regulation of innate immune response | ERK1 and ERK2 cascade |
| 22 | alcohol metabolic process | regulation of amide metabolic process | regulation of actin filament-based process |
| 23 | vesicle organization | regulation of cytokine-mediated signaling pathway | regulation of cellular component size |
| 24 | phospholipid metabolic process | cytosolic pattern recognition receptor signaling pathway | calcium ion transport |
| 25 | positive regulation of protein kinase activity | regulation of response to cytokine stimulus | regulation of neurogenesis |
| 26 | protein localization to cell periphery | nucleobase-containing compound transport | regulation of viral genome replication |
| 27 | small GTPase mediated signal transduction | protein stabilization | cellular response to UV |
| 28 | defense response to bacterium | regulation of protein ubiquitination | nerve development |
| 29 | regulation of protein-containing complex assembly | meiotic nuclear division | cellular response to light stimulus |
| 30 | positive regulation of cell projection organization | glycerolipid biosynthetic process | viral genome replication |
| 31 | negative regulation of protein modification process | meiotic cell cycle process | regulation of epithelial cell apoptotic process |
| 32 | establishment of organelle localization | regulation of protein modification by small protein conjugation or removal | response to toxic substance |
| 33 | establishment of vesicle localization | negative regulation of growth | release of sequestered calcium ion into cytosol |
| 34 | vesicle localization | mitotic nuclear division | negative regulation of sequestering of calcium ion |
| 35 | epidermal cell differentiation | developmental cell growth | regulation of sequestering of calcium ion |
| 36 | stress-activated protein kinase signaling cascade | regulation of post-translational protein modification | sequestering of calcium ion |
| 37 | negative regulation of growth | RNA splicing, via transesterification reactions | stem cell proliferation |
| 38 | glycerophospholipid metabolic process | RNA splicing, via transesterification reactions with bulged adenosine as nucleophile | regulation of viral life cycle |
| 39 | protein targeting | mRNA splicing, via spliceosome | leukocyte apoptotic process |
| 40 | canonical Wnt signaling pathway | regulation of protein stability | epithelial cell apoptotic process |

**Methods and Materials**

***Animal experiments***

All animal experiments were conducted according to the animal experimentation regulations of Germany and were approved by the Ethics Committee of the Regional Council Freiburg. All mice derive from the C56BL/6 strain and were bred at the central animal facility of the University of Konstanz. All mice were held in groups of 2-6 animals in individually ventilated cages in 12:12 h light/dark cycle with permanent access to water and standard chow diet *ad libitum*. Sample sizes for animal studies were estimated from previous studies or determined using G*Power (3.1.9.2) with alpha set to 0.05 and a power level to 0.95. K14-CreER^Tam^Cyp11b1^L2/L2^ and Cyp11b1^L2/L2^ mice were generated as described before ^1^. All *in vivo* experiments were conducted using sex-matched littermate mice that were between 7 and 14 weeks old.

Keratinocyte-specific deletion of *Cyp11b1* was induced by treating mice with tamoxifen for five consecutive days, either by intra-peritoneal (i.p.) injection, here termed as L2/L2 or KO with intact barrier (IntB), or by topical application, here termed as L2/L2 or KO with mild barrier disruption (BD). For i.p. administration, mice were daily i.p. injected with 100 µl tamoxifen (Sigma-Aldrich) in ethanol-oil solution (10 mg/ml, 1:8 ethanol:oil). For topical application, back hair was clipped, and tamoxifen, dissolved in ethanol (25 mg/ml), was topically applied on dorsal skin (100 µl) and ears (20 µl) for five consecutive days. All animals were sacrificed one day (Day 6) or nine days (Day 14) after the last tamoxifen treatment.

***Genotyping***

Genomic DNA was purified from mouse tail biopsies and lung tissue with the SV Total Genomic DNA isolation kit (Promega) according to the manufacturer’s protocol. PCR amplification (35 cycles: 94°C, 62°C and 72°C, 30 sec each step) was performed to detect the Cyp11b1 deletion product (Fw: TGC AGC ATG CTC ATG CAT TGG G, Rev: CTG AGA CAG GCA GGG ATC ATG TCT CAG), the CreERTam fusion gene (Fw: GAA CCT GAT GGA CAT GTT CAG G, Rev: AGT GCG TTC GAA CGC TAG AGC CTG T) and myogenin (Fw: TTA CGT CCA TCG TGG ACA GC, Rev: TGG GCT GGG TGT TAG CCT TA). PCR products were separated by gel electrophoresis and visualized using ImageQuantLAS400 (GE Healthcare Life Sciences).

***Tissue ex vivo culture and luciferase-based glucocorticoid reporter assay***

Dorsal skin was isolated and subcutaneous fat was removed before excising 8 mm diameter biopsy punches. Biopsies were incubated by floating on serum-free DMEM (Sigma-Aldrich) medium with or without 300 µg/ml metyrapone (MET) for overnight. Supernatants were collected, boiled (10 min, 95°C), centrifuged (15 min, full speed, 4 °C) and used for the luciferase reporter-based GC bioassay. Corticosterone levels in serum (diluted 1:100) and *ex vivo* skin cultures (undiluted) were determined by a previously published luciferase-based GC bioassay ^1^. In short, serum and *ex vivo* culture supernatant were added to HEK293T cells transiently transfected with a glucocorticoid receptor (GR) expression plasmid, a GC response element (GRE)-containing luciferase reporter construct and a β-galactosidase expression plasmid for normalization. Luciferase activity was measured after overnight incubation and corticosterone concentrations in serum samples were calculated using a standard curve. For skin *ex vivo* samples, corticosterone levels measured in MET-treated samples were subtracted from untreated samples to exclude contamination by serum-derived GC. The values are displayed as fold change over the mean of untreated samples of control mice.

**S*andwich ELISA***

Interleukin (IL)-6 concentrations in serum (diluted 1:2) were quantified by ELISA according to the manufacturer’s protocol (Invitrogen).

***Isolation of skin and draining lymph node immune cells***

Immune cells from ear skin and draining lymph nodes (dLNs) for subsequent flow cytometry analysis were isolated as described before ^1^. Briefly, ears were manually separated and digested in serum-free DMEM (Sigma-Aldrich) containing 0.25 mg/ml Liberase Thermolysin Low (Roche) for 1.5 hours at 37 °C. Single cell suspension was obtained by filtering through 70 µm strainer. Axillary and auricular dLNs were perforated with 26-gauge syringe needles and digested for 30 min at 37 °C in digestion solution (DMEM, 10% FBS, collagenase IV (0.75 mg/ml) (Sigma-Aldrich), deoxyribonuclease I (40 µg/ml) (Roche), 3 mM CaCl_2_), before digestion was stopped with 0.5 M EDTA. Skin and dLN-derived cells were filtered with 70 µm strainers and counted using trypan blue staining prior to processing for flow cytometry.

***Flow cytometry***

Immune cell phenotyping was performed by high-dimensional flow cytometry as described before ^1,2^. For skin and dLN analysis at day 6 with focus on mononuclear phagocytes, isolated cells from skin and dLNs were stained 1:1000 with Fixable Viability Dye (FVD455UV) (eBioscience) for 30 min at 4 °C, washed with FACS Buffer (2% BSA, 2 mM EDTA), blocked in 1:200 anti-CD16/32 TruStain Fcγ PLUS antibody (BioLegend) and 3% BSA for 30 min at 4 °C, stained with a combination of surface marker antibodies that are labeled with fluorophores (Supplementary table S1) for 45 min at 4 °C, and washed with cold PBS prior to measuring at the LSR Fortessa analyzer (BD).

For dLN analysis at day 14 with focus on lymphoid cells and their expression of IL-17A and IFN-γ, isolated dLN cells were re-stimulated for 5 hours at 37°C with 50 ng/mL PMA, 500 ng/mL Ionomycin and 10 µg/mL Brefeldin A, followed by a viability dye staining using FVD455UV, blocking, and staining with surface antibodies (Supplementary table S2) as described above. To assess their activation state, cells were thereafter intracellularly stained by permeabilization and fixation with Foxp3/Transcription Factor Staining Buffer Set (eBioscience), followed by intracellular blocking as described, and staining with cytokine-specific antibodies for overnight at 4°C (Supplementary table S2). On the next day, cells were washed in cold PBS and measured at LSR Fortessa (BD).

***Flow cytometry analysis***

Flow cytometry data was preprocessed with FlowJo (10.8.1, TreeStar) to export compensation-corrected, debris-cleaned, live CD45+ single cells. FCS files were imported and analyzed in R environment (version 4.3.2) using described workflows for unsupervised and automated cluster identification ^3–5^. In brief, FCS data was transformed via inverse hyperbolic arcsinh function with individually set cofactors for single markers. Surface marker expression was then percentile-normalized to values between 0 and 1. Uniform Manifold Approximation and Projection (UMAP) plots were generated to visualize single-cell data using umap R package (version 0.2.8.0) and cell clusters were generated using FlowSOM (version 2.0.0) and ConsensusClusterPlus (version 1.56.0) packages and manually merged based on specific marker expression in order to attribute them to different immune cell subsets. Frequencies and total cell numbers, calculated from counted viable cells, of the different cell populations were exported and visualized.

***Histology – H&E staining from cyro-sections***

Mouse ears were embedded in Tissue-Tek O.C.T (Sakura) and frozen using dry ice. Cryosections (8 µm thickness) on microscope slides were fixed with ice-cold acetone for 10 min, submerged into deionized water to remove the cryo-embedding medium, and subsequently stained with hematoxylin and eosin (H&E).

***Keratinocyte isolation and RNA extraction***

Keratinocytes from dorsal skin and ear skin were isolated and pooled for subsequent RNA extraction and sequencing. Dorsal skin was gently shaved and removed from sacrificed mice. After subcutaneous fat removal, skin was cut into small pieces (0.5 x 1 cm) and together with extracted ear skin washed in cold PBS supplemented with antibiotics. Skin pieces were then incubated in 2.4 U/ml Dispase II (Sigma-Aldrich) in serum-free DMEM for 1.5 hours at 37 °C. Afterwards, the epidermis was peeled off and placed on 1 ml drops of Accutase® solution (Sigma-Aldrich) and incubated for 15 min at 37°C. Mechanical mixing and rubbing using curved forces facilitated the release of epidermal cells. Cell suspension was filtered through 40 µm strainer and centrifuged at 300 x g for 6 min, 4 °C. Cells were resuspended in DMEM media, counted, and transferred into V-bottom plate with 4-5 x10^6^ cells per well. Cells were blocked as described in the flow cytometry protocol, and stained with anti-CD45-FITC (eBioscience), anti-CD49f-PE (BioLegend), and anti-CD34-APC (BioLegend) antibody for 45 min, 4 °C. After washing twice with cold PBS, cells were resuspended in sort buffer (PBS, 1 mM EDTA, 25 mM HEPES pH 7, 1% BSA), stained with Sytox™ Blue (Thermo Fisher) to exclude dead cells, filtered through 30 µm strainer and sorted using FACSAria III Sorter (BD). Live keratinocytes were gated for CD45-, CD49f+ and CD34+ cells and bulk-sorted for downstream RNA extraction (see supplementary figure 2).

Total RNA of 2 x10^6^ sorted keratinocytes per sample (mouse) was extracted using the RNeasy Plus Mini Kit (QIAGEN) according to manufacturer’s instructions.

***RNA sequencing***

RNA sequencing (single-end 100 bp) of keratinocyte total RNA, including quality control, Illumina’s SmartSeq II library construction and cluster generation, was performed at the Functional Genomics Center Zurich with NovaSeq 6000 (Illumina Inc.) according to standard protocols as described previously ^6^.

Raw reads were aligned to mouse genome (GRCm38) using STAR aligner and translated into read counts using FeatureCounts based on GENCODE gene annotation version M16.

***RNA sequencing analysis***

The count matrix was exported and cleaned from genes with low counts (>=10), mitochondrial, and ribosomal genes and imported in R studio (version 4.3.2) and further analyzed in R environment. Gene counts were normalized and log-transformed and DEGs were determined using DESeq2 (version 1.42.0) package with an adjusted p-value <0.05. Principal component analysis (PCA) plots were generated with limma (version 3.58.1) package. Heatmaps and Volcano plots were generated using “EnhancedVolcano” (version 1.20.0) and pheatmap (version 1.0.12) packages with a cut-off of Log_2_ fold change >1, respectively <-1 and p-value <0.05. Pathway analysis was performed using Progeny (version 1.24.0) package. For gene ontology (GO) analysis on biological processes (BP), normalized counts were aligned to the murine Ensembl data base using biomaRt (version 2.58.2) package and analyzed with clusterProfiler (version 4.10.0) and enrichplot (version 1.22.0).

***Statistical analysis and data presentation***

Statistical differences were determined using GraphPad Prism software (v8) or in R environment. Significance level alpha was set to 0.05 for all statistical analyses with *p < 0.05, **p < 0.01, ***p < 0.001, and ****p < 0.001. Details of each statistical test applied on the data are indicated in the corresponding figure legend. If not stated otherwise, data are mean +/- standard deviation (SD) of a minimum of five mice per group.

***References***

1. Phan TS, Schink L, Mann J, et al. Keratinocytes control skin immune homeostasis through de novo–synthesized glucocorticoids. *Sci Adv*. 2021;7(5):1-20. doi:10.1126/sciadv.abe0337

2. Lambrecht R, Delgado ME, Gloe V, et al. Liver receptor homolog-1 (NR5A2) orchestrates hepatic inflammation and TNF-induced cell death. *Cell Rep*. 2023;42(12):113513. doi:10.1016/j.celrep.2023.113513

3. Nowicka M, Krieg C, Crowell HL, et al. CyTOF workflow: differential discovery in high-throughput high-dimensional cytometry datasets. *F1000Research*. 2019;6:748. doi:10.12688/f1000research.11622.3

4. Brummelman J, Haftmann C, Núñez NG, et al. Development, application and computational analysis of high-dimensional fluorescent antibody panels for single-cell flow cytometry. *Nat Protoc*. 2019;14(7):1946-1969. doi:10.1038/s41596-019-0166-2

5. Ingelfinger F, Krishnarajah S, Kramer M, et al. Single-cell profiling of myasthenia gravis identifies a pathogenic T cell signature. *Acta Neuropathol*. 2021;141(6):901-915. doi:10.1007/s00401-021-02299-y

6. Zwicky P, Ingelfinger F, de Melo BMS, et al. IL-12 regulates type 3 immunity through interfollicular keratinocytes in psoriasiform inflammation. *Sci Immunol*. 2021;6(64):eabg9012. doi:10.1126/sciimmunol.abg9012
